# A Nationwide Population-Based Cohort Study: Will Anxiety Disorders Increase Subsequent Cancer Risk?

**DOI:** 10.1371/journal.pone.0036370

**Published:** 2012-04-27

**Authors:** Ji-An Liang, Li-Min Sun, Kuan-Pin Su, Shih-Ni Chang, Fung-Chang Sung, Chih-Hsin Muo, Chia-Hung Kao

**Affiliations:** 1 Department of Radiation Oncology, China Medical University Hospital, Taichung, Taiwan; 2 Institute of Clinical Medicine Science and School of Medicine, College of Medicine, China Medical University, Taichung, Taiwan; 3 Department of Radiation Oncology, Zuoying Armed Forces General Hospital, Kaohsiung, Taiwan; 4 Department of General Psychiatry & Mind-Body Interface Laboratory, China Medical University Hospital, Taichung, Taiwan; 5 Management Office for Health Data, China Medical University Hospital, Taichung, Taiwan; 6 Institute of Environmental Health, College of Public Health, China Medical University, Taichung, Taiwan; 7 The Ph.D. Program for Cancer Biology and Drug Discovery, China Medical University, Taichung, Taiwan; 8 Institute of Biomedical Sciences, Academia Sinica, Taipei, Taiwan; 9 Department of Nuclear Medicine and PET Center, China Medical University Hospital, Taichung, Taiwan; University of California Irvine, United States of America

## Abstract

**Background:**

The aim of this study was to evaluate a possible association between malignancy and anxiety disorders (AD) in Taiwan.

**Methods:**

We employed data from the National Health Insurance system of Taiwan. The AD cohort contained 24,066 patients with each patient randomly frequency matched according to age and sex with 4 individuals from the general population without AD. Cox's proportional hazard regression analysis was conducted to estimate the influence of AD on the risk of cancer.

**Results:**

Among patients with AD, the overall risk of developing cancer was only 1% higher than among subjects without AD, and the difference was not significant (hazard ratio [HR] = 1.01, 95% confidence interval [95% CI] = 0.95–1.07). With regard to individual types of cancer, the risk of developing prostate cancer among male patients with AD was significantly higher (HR = 1.32, 95% CI = 1.02–1.71). On the other hand, the risk of cervical cancer among female patients with AD was marginally significantly lower than among female subjects without AD (HR = 0.72, 95% CI = 0.51–1.03).

**Limitations:**

One major limitation is the lack of information regarding the life style or behavior of patients in the NHI database, such as smoking and alcohol consumption.

**Conclusions:**

Despite the failure to identify a relationship between AD and the overall risk of cancer, we found that Taiwanese patients with AD had a higher risk of developing prostate cancer and a lower risk of developing cervical cancer.

## Introduction

Anxiety disorder (AD) is a chronic disorder, highly prevalent in the adult population, with the lifetime prevalence in the general population estimated at 5% in western countries [1], [2]. Studies in Asia have shown an even higher lifetime prevalence [3]. In a study in Taiwan, Hwu et al. [4] found that the life time prevalence of AD differed considerably according to geographic sampling area, ranging from 3.7% to 10.5%. AD often causes sleep disturbance [5], and has a detrimental effect on health outcomes such as increased risk of heart disease and respiratory resistance in asthma [6], [7].

In examining the relationship between AD and malignancy, most studies have focused on the issue of anxiety among patients with malignancy and found that increased anxiety is not uncommon among cancer patients [8]–[10]. However, in this study, we explored the possibility of reverse causality, to determine whether AD is a risk or protective factor in the development of malignancy. Following a review of the literature, we found that only a limited number of papers discussed or even addressed this issue, and the association between AD and cancer remains unclear [11]–[15]. Recently published meta-analysis focusing only on prospective studies uncovered the fact that psychological distress, including depression, anxiety, and poor quality of life, are associated with a statistically significant (6%) increase in the risk of cancer [16].

To the best of our knowledge, no nationwide, population-based studies outlining the possible relationship between AD and cancer have been conducted in Taiwan. The aim of this study was to assess the risk of all forms of cancer among patients with AD in Taiwan. The results, presented in this paper, were generated from a retrospective cohort study of patients with AD. The original database was derived from the National Health Insurance (NHI) system in Taiwan.

## Methods

### Database

This study used claims data from the National Health Insurance Research Database (NHIRD), retrospectively collected from NHI, representing health care data from >96% of all medical claims in Taiwan since 1996. The NHI program provides comprehensive medical services, including ambulatory care, inpatient care, physical therapy, dental services, prescription drugs, medical institutions, and registration files with scrambled identifications. We obtained a representative claims dataset of a sample of one million enrollees, randomly selected from the entire insured population included in the NHI program base between 1996 and 2000. The diagnoses in the database were coded using the International Classification of Diseases 9th Revision of the Clinical Modification (ICD-9-CM). A more detailed description of the NHIRD was previously described [17].

### Study sample

For the study cohort, we selected patients who had been diagnosed with AD between 1 January 2000 and 31 December 2002 (ICD-9-CM codes 300.0, 300.2, 300.3, 308.3 and 309.81). To ensure the accuracy of diagnosis of AD, we selected only subjects who had been admitted at least three times as a study cohort. We then excluded patients who had any type of cancer (ICD-9-CM codes 140–239) prior to the date of indexing. Finally, we identified a total of 24,066 patients with AD. To assemble a comparison cohort, we randomly selected 96,104 enrollees without a history of AD. These controls were matched with the study cohort at a ratio of 1∶4 on frequency of age and sex using the same exclusion criteria during the same period.

Both study cohorts were followed until a diagnosis of cancer was made, or until the time the subject was lost to follow-up, death, termination of insurance, or other causes by the end of 2008.

In addition, we also searched hypertension (ICD-9-CM codes 401–405) and diabetes mellitus (ICD-9-CM codes 150), and hyperlipidemia (ICD-9-CM codes 272) as comorbidities at the baseline.

### Statistical analysis

A chi-square tests were carried out to examine the differences in the distribution of socio-demographic factors and comorbidities between the cohorts with and without AD. Person-years of follow-up duration were calculated for each person until cancer was diagnosed or censored. A Poisson regression model was used to examine the incidence density rates and incidence rate ratio (IRR) and 95% confidence interval (95% CI) of cancer with categorical variables were calculated for each cohort. Multivariate Cox proportional hazard models were used to estimate hazard ratio (HR) and 95% CI for factors associated with cancer risk. We performed all analysis using the SAS statistical package (SAS System for Windows, Version 9.1) with the statistical adopted significance level at 0.05.

## Results

Of the total sample of 120,170 subjects, with a mean age of 54.0 years in the non-AD cohort and 54.1 years in the AD cohort. When focused on AD group only, we found the AD cohort tended to comprise more females (64.3%) and was more likely to be younger (26.9% in 30–44 years of age). Compared to the non-AD group, the AD cohort tended to live in lower urbanized areas (28.9% in urbanization level 4 and 5), and to have hypertension (44.6% vs. 26.7%), diabetes mellitus (11.6% vs. 9.5%) and hyperlipidemia (24.7% vs. 12.9%) (Table 1).

**Table 1:** Baseline characteristics between anxiety disorder group and non-anxiety disorder group in 2000–2002.

Variables	Anxiety disorder				
	No N = 96,104		Yes N = 24,066		
	n	%	n	%	p-value
Sex					0.86
Women	61,768	64.3	15,482	64.3	
Men	34,336	35.7	8,584	35.7	
Age, years					1.00
30–44	29,292	27.0	7,323	26.9	
45–54	24,764	22.8	6,191	22.8	
55–64	18,029	16.6	4,524	16.6	
≥65	24,019	22.1	6,028	22.2	
Mean (SD)†	54.0	(14.3)	54.1	(13.9)	0.94
Urbanization level					<0.0001
1	28,742	29.9	6,374	26.5	
2	27,506	28.6	6,785	28.2	
3	16,749	17.4	3,959	16.5	
4	12,966	13.5	4,020	16.7	
5	10,140	10.6	2,928	12.2	
Co-morbidity					
Hypertension	25,678	26.7	10,732	44.6	<0.0001
Diabetes mellitus	9,089	9.5	2,798	11.6	<0.0001
Hyperlipidemia	12,401	12.9	5,932	24.7	<0.0001

Urbanization level: 1 indicate the highest level of urbanization and 5 the lowest

Chi-square test,

† student T-test.

Hypertension: ICD-9-CM (A-code): 401–405 (A260 and A269), admission more than three times.

Diabetes mellitus: ICD-9-CM (A-code): 250 (A181), admission more than twice in the first year.

Hyperlipidemia: ICD-9-CM (A-code): 272 (A182), admission more than three times.

Table 2 presents a comparison of incidence density of cancer during the 9-year follow-up period for subjects with and without AD, according to socio-demographic factors and comorbidities. The incidence of cancer was slightly higher in the AD cohort than in the non-AD cohort (7.59 vs. 7.33 per 1,000 person-years), with an IRR of 1.04 (95% CI = 0.98–1.10). The IRR of cancer was highest in the youngest age group (30–44 years of age, IRR = 1.18, 95% CI = 0.99–1.40). Among the patients with hypertension, we observed that patients with AD had a lower incidence than subjects without AD (10.3 vs. 11.9 per 1,000 person-years; IRR = 0.87, 95% CI = 0.80–0.94).

**Table 2:** Comparisons of incidence density of cancer between anxiety disorder group and non-anxiety disorder group by characteristics.

	Anxiety disorder							
	No			Yes				
Variables	Cases	Person-years	Rate†	Cases	Person-years	Rate†	IRR	(95% CI)
All	5,503	750,350	7.33	1,467	193,207	7.59	1.04	(0.98–1.10)
Sex								
Women	2,994	490,473	6.10	801	126,440	6.34	1.04	(0.96–1.12)
Men	2,509	259,877	9.65	666	66,767	9.97	1.03	(0.95–1.13)
Age, years								
30–44	563	240,916	2.34	169	61,461	2.75	1.18	(0.99–1.40)
45–54	1,087	204,396	5.32	290	51,421	5.64	1.06	(0.93–1.21)
55–64	1,298	144,457	8.99	341	36,672	9.30	1.03	(0.92–1.17)
≥65	2,555	160,581	15.9	667	43,653	15.3	0.96	(0.88–1.05)
Urbanization level								
1	1,502	227,091	6.61	364	51,455	7.07	1.07	(0.96–1.20)
2	1,562	216,528	7.21	388	54,886	7.07	0.98	(0.88–1.10)
3	906	130,615	6.94	228	31,686	7.20	1.04	(0.90–1.20)
4	851	99,830	8.52	269	32,156	8.37	0.98	(0.86–1.13)
5	682	76,276	8.94	218	23,024	9.47	1.06	(0.91–1.23)
Co-morbidity								
Hypertension								
No	3,314	565,762	5.86	609	109,651	5.55	0.95	(0.87–1.03)
Yes	2,189	184,589	11.9	858	83,556	10.3	**0.87**	**(0.80–0.94)** **
Diabetes mellitus								
No	4,733	686,830	6.89	1,223	171,974	7.11	1.03	(0.97–1.10)
Yes	770	63,520	12.1	244	21,232	11.5	0.95	(0.82–1.09)
Hyperlipidemia								
No	4,609	654,514	7.04	1,060	145,678	7.28	1.03	(0.97–1.10)
Yes	894	95,836	9.33	407	47,528	8.56	0.92	(0.82–1.03)

† per 1,000 person-year.

IRR: incidence rate ratio.

* p<0.05,

** p<0.001.

Multivariate analysis with Cox proportional hazard regression showed that after adjusting for the urbanization level and comorbidities, patients with AD was not significantly related to the development of cancer (HR = 1.01, 95% CI = 0.95–1.07) (Table 3).

**Table 3:** Hazard ratios and 95% confidence interval of cancer associated with anxiety disorder in Cox's regression analysis in different cancer.

	Percentage of cancer			
Variables	Non- Anxiety disorder	Anxiety disorder	HR	(95% CI)
Overall	5.73	6.10	1.01	(0.95–1.07)
Oral cancer	0.26	0.26	0.81	(0.40–1.61)
Colorectal cancer	0.86	0.84	0.92	(0.79–1.08)
Liver cancer	0.72	0.85	1.11	(0.95–1.30)
Lung cancer	0.74	0.81	1.05	(0.89–1.23)
Breast cancer (women only)	0.98	1.16	1.32	(0.95–1.34)
Cervical cancer (women only)	0.35	0.25	0.72	(0.51–1.03)**
Prostate cancer (men only)	0.66	1.00	**1.32**	**(1.02–1.71)** *
Other cancers	2.06	2.08	1.00	(0.88–1.15)

ICD-9-CM: oral cancer, 140.xx–149.xx; colorectal cancer, 153.xx and 154.xx; liver cancer, 155.xx; lung cancer, 162.xx; breast cancer, 174.xx; cervical cancer, 180.xx; prostate cancer, 185.xx.

Adjusted for urbanization, hypertension, diabetes mellitus, and hyperlipidemia.

* p-value<0.05,

** p-value = 0.0678.

However, further specific analysis on cancer type determined that patients with AD had a significantly higher risk of prostate cancer (HR = 1.32, 95% CI = 1.02–1.71) (Table 3); particularly in the older age group (≥65 years; HR = 1.45, 95% CI = 1.09–1.93) (Table 4). In addition, we also observed that the AD cohort had a significantly higher percentage than the non-AD cohort to undergo prostate specific antigen (PSA) testing, regardless of the age group (30–44 years, 7.83% vs. 2.92%; 45–54 years 32.0% vs. 12.1; 55–64 years, 46.2% vs. 25.2%; ≥65 years, 54.5% vs. 35.9%) (Table 5). Table 3 also revealed that patients with AD had a marginally significantly lower risk to develop cervical cancer (HR = 0.72, 95% CI = 0.51–1.03), and Table 5 showed a significantly higher percentage of AD cohort to undergo Papanicolaous smear testing compared to non-AD cohort.

**Table 4:** Multivariable Cox's regression analysis of cancer associated with anxiety disorder among age groups.

	Percentage of cancer			
Variables	Non-Anxiety disorder	Anxiety disorder	HR	(95% CI)
Prostate cancer (men only)				
30–44	0.00	0.00	–	
45–54	0.16	0.10	0.46	(0.10–2.14)
55–64	0.75	0.98	1.25	(0.68–2.29)
≥65	1.63	2.66	**1.45**	**(1.09–1.93)** *
Cervical cancer (women only)				
30–44	0.21	0.06	0.33	(0.10–1.06)
45–54	0.34	0.35	0.98	(0.55–1.76)
55–64	0.42	0.30	0.78	(0.38–1.60)
≥65	0.50	0.32	0.67	(0.35–1.28)

Adjusted for urbanization, hypertension, diabetes mellitus, and hyperlipidemia.

* p<0.05,

** p<0.001,

*** p<0.0001.

**Table 5:** The distribution of prostate specific antigen and Papanicolaous smear between anxiety disorder group and non-anxiety disorder group.

	Anxiety disorder				
	No		Yes		
Variable	n	%	n	(%)	p-value
PSA					
Age, year					
30–44	294	2.92	197	7.83	<0.0001
45–54	939	12.1	620	32.0	<0.0001
55–64	1,546	25.2	709	46.2	<0.0001
≥65	3,730	35.9	1,417	54.5	<0.0001
Papanicolaous smear					
Age, year					
30–44	3,868	20.1	1,586	33.0	<0.0001
45–54	3,133	18.4	1,334	31.3	<0.0001
55–64	1,452	12.2	689	23.1	<0.0001
≥65	1,025	7.52	475	13.9	<0.0001

PSA: prostate specific antigen.

chi-square test.

## Discussion

In this population-based cohort study, the main finding was the lack of an association between AD and the overall risk of cancer in Taiwan; however, we obtained a number of interesting findings related to the individual risk of cancer. Males with AD 65 years old and older had a significantly higher risk of developing prostate cancer. In contrast, females with AD had a marginally significantly lower risk of developing cervical cancer.

As of 1982, cancer has been the leading cause of death among the general population in Taiwan. The age-adjusted incidence rate has increased steadily reaching 270 new cases per 100,000 in 2007 [18]. The trend is different from Surveillance Epidemiology and End Results data, which showed that the overall incidence rates of cancer for all racial/ethnic groups combined decreased by 0.7% per year between 1999 and 2006 [19]. Because this issue remains a challenge for the public health system in Taiwan, it has come to the attention of the government, resulting in a population-based investigation regarding cancer epidemiology and preventive. The Taiwanese NHI health insurance program is a good resource providing valuable materials with which to approach populated-based studies. We recently used it to evaluate the risk of malignancy for patients with systemic lupus erythematosus, resulting in positive findings [20]. The current study used a similar design in an attempt to identify a relationship between AD and the risk of cancer.

To the best of our knowledge, this is the first population-based study with a collection of 24,066 patients with AD in Taiwan. To create a control group, we randomly frequency matched each AD patient with 4 individuals from the general population without AD but in a similar age, sex, and index calendar year. To compare demographic characteristics between the two groups, our data shows that patients with AD tended to have comorbidities. This is expected because chronic illness may induce AD, and vice versa [6], [21], [22]. The distribution of urbanization levels illustrated a significantly higher prevalence of AD patients living in less urbanized areas of Taiwan. This is consistent with a prior study from Taiwan, indicating that individuals living in rural areas had a higher risk of AD [4].

The potential relationship between AD and the overall risk of cancer or any individual risk of cancer is still not well-recognized. Although prior meta-analysis found a significant increase in the overall risk of cancer for patients with psychological distress [16], only a non-significant 1% increase in the overall risk of cancer risk was observed in our study. With regard to the individual risk of cancer, we analyzed the most common types of cancer in Taiwan and the results show different directions for various forms of cancer. However, only prostate cancer showed a significantly different risk between AD and non-AD groups. Most previous studies have focused on psychological factors and breast cancer, and the results have been contradictory. Some studies showed an increased risk of breast cancer [23], [24], while such associations were not observed in other studies [13], [25]. Earlier meta-analysis found only a modest association between specific psychosocial factors and breast cancer, contrary to the conventional wisdom that personality and stress influence the development of breast cancer [26]. Our study showed a 32% increase in the risk of breast cancer among patients with AD; however, the difference did not reach statistical significance.

Our results show a significant increase in the risk of developing prostate cancer, particularly in the older age group. This is a novel finding not uncovered in previous studies [16]. One possible reason is that patients with AD tend to check their health more regularly, and are assumed to get routine check-ups, including cancer screening more frequently than the general population. Table 5 validates our assumption showing that patients with AD consistently have significantly higher PSA rates among various age groups. This may result in an increase in the early detection of prostate cancer. The same explanation may also be implied for patients with cervical cancer. We found a marginally significant decrease (28%) in the incidence rate for invasive cervical cancer among female patients with AD. More frequent Pap smear exams could identify more patients with cervical CIS/CIN/dysplasia. CIN and CIS lesions are known to precede invasive cancer in the process of carcinogenesis for cervical cancer. Normally, these patients receive treatment before the disease progresses into invasive cancer. We did not include CIN/CIS as our end-point for cervical cancer and this could decrease the relative number of cases of invasive cervical cancer. The data from the table 5 further supports this assumption. Table 1 revealed significant difference for the distribution of urbanization levels between AD and non-AD cohorts, and people live in the areas of higher urbanization levels may have more frequently screened for cancer; however, due to the limitation of the database, it is difficult to verify from this study.

Psychological parameters could alter immune and endocrine function, and it has long been hypothesized that, through this pathway, psychosocial factors may influence the incidence of cancer [27]. A growing body of evidence has implicated the immune system as a link between the central nervous system and the processes of disease [28]. A number of researchers in this field have proven that external factors such as stress, depression, or social support have a significant influence on the immune system, which in turn influence the onset and/or the course of cancer [29].

The strength of this study is the population-based design with its generalizability. However, one major limitation that needs to be addressed is the lack of information regarding the life style or behavior of patients in the NHI database, making it impossible to adjust for health behavior related factors such as smoking and alcohol consumption. Specific personality traits and various life events are associated with health-related behavior and lifestyle factors, e.g., smoking or unhealthy nutrition. These unhealthy habits can increase the risk of cancer [27]; however, the relationship between health behavior and AD remains controversial [30], [31].

In conclusion, this population-based retrospective cohort study identified a significant increase in the incidence of prostate cancer risk and a marginally significant decrease in the risk of cervical cancer among Taiwanese patients with AD. The intrinsic characteristic of patients with AD cause them to seek cancer screening more frequently, and this fact contributes to the results. The findings in this study may remind the NHI of Taiwan to reconsider policy regarding follow-up and cancer screening in patients with AD.
